# Response to “Tailored Use of Avacopan in a Case With Refractory Antineutrophil Cytoplasmic Autoantibody-Associated Renal Vasculitis and Concominant Complement System Activation”

**DOI:** 10.1016/j.ekir.2022.11.014

**Published:** 2022-11-25

**Authors:** Jolijn R. van Leeuwen, Ton J. Rabelink, Y.K. Onno Teng

**Affiliations:** 1 Center of Expertise for Lupus, Vasculitis- and Complement-mediated Systemic diseases, Department of Internal Medicine—Nephrology Section, Leiden University Medical Center, Leiden, The Netherlands

The Author Replies:

We appreciate the interest from Hakroush and Tampe in our manuscript describing compassionate use of avacopan in 8 difficult-to-treat patients with antineutrophil cytoplasmic autoantibody-associated vasculitis (AAV).^1^ In their letter, the authors present a very interesting case of avacopan treatment in a refractory AAV patient with renal involvement.

The disease course of the patient described by the authors is in line with the disease course of 6 refractory AAV patients we previously described.^1^ All patients had ongoing vasculitis activity after remission with conventional induction treatments, but reached remission shortly after the start of avacopan. The presented case, however, is unique in its observation that dialysis-dependency resolved quickly after initiating avacopan. This case emphasizes the value of real-world data, which suggest avacopan might be beneficial for patients with refractory disease.

In the era of big data analyses, it is important to appreciate that case-reports and case-series remain pivotal as hypothesis-generating clinical observations. In contrast to the pivotal randomized ADVOCATE trial that produced evidence for a steroid-free treatment strategy for active AAV patients,^2^ the indication for avacopan in refractory patients is not yet clear. In addition to the unfamiliarity of physicians with this new drugs and the accompanying high drug costs of avacopan, guideline recommendations are still unclear on the place of avacopan in the treatment strategies for AAV. In the present setting, it is important to identify those AAV patients who would benefit the most from avacopan and it emphasizes the lack of an adequate biomarker predicting response or long-term remission for the use of avacopan. The authors postulate that local complement activation at the kidney tissue level might be such a potential biomarker but this requires formal validation in a larger setting. Previous studies have corroborated that low C3 serum levels are associated with worse outcomes in patient with renal involvement and are a predictor of treatment resistance in pauci-immune glomerulonephritis,^3^^,^^4^ although most often, serum complement levels in AAV patients are normal.^4^

In summary, the unique and beneficial clinical observation described in the case by Hakroush and Tampe should prompt clinicians to collaborate in collecting and describing the real-world experience of treating in a low-prevalent AAV patients with a novel complement-targeting drug avacopan.
